# Alcohol abuse patients lack follow up possibilities following ED discharge

**DOI:** 10.1186/1757-7241-23-S1-A38

**Published:** 2015-07-16

**Authors:** Pernille Würtz Bøhm RN

**Affiliations:** 1 The Emergency Department, Holbaek University Hospital, Holbaek, Denmark

## 

Alcohol is the major involvement for people seeking help at Emergency Departments (ED). In addition, it is demanding for the health professionals to talk with patients about alcohol abuse. The aim for this study was to examine current practices of treating alcohol abuse in the Quick Diagnostic Unit (QDU).

## Methods

A qualitative semi-structured interview of nine hospitalized patients in a QDU. Patients were interviewed regarding social background, alcohol use, motivation to stop drinking, and the treatment in the QDU, their personal needs while hospitalized and upon discharge.

A questionnaire was sent to all ED nurses. The nurses were asked about their priority of the patients during their daily shift of 7.5 hours. The questions were divided into two groups, "non-alcohol abusers" and "alcohol abusers". The nurses were asked to rate nursing requirement.

## Results

Patients were not hospitalized to get alcohol detoxification but because they were frightened for their health. The patients' drinking habits were beyond the Danish drinking average (Statistics Denmark, Consumption of alcohol and tobacco 2011, Published in News of Statistics Denmark), which is 11 units of alcohol per week. Average drinking for the patients at the QDU was 19 units per day (± 6.5). The reported alcohol use was 228 gram per day (± 36). Patients requested more information about their body's biochemical status. All patients knew they were not healthy, and they pointed out the information regarding their wellbeing as a central key for their alcohol habits.

Five patients had psychological diagnoses and three said they had psychological issues that had a bad influence on daily living.

Nurses spent one hour less on each shift on patients with alcohol abuse compared with non-alcohol abusers. 73% of the nurses experienced that patients with mental disorders did not get the sufficient treatment after discharge. 43% of the nurses did not think that the estimated time used on patients with alcohol abuse enhanced patients' health.

## Conclusion

Alcohol abusers were hospitalized due to acute health problems. They needed more information while hospitalized. And they were heavy drinkers. The nurses spent less time with the group of patients with alcohol abuse and it did not improve their health. The primary sector should focus on the discharge offers.

